# Aging and Type 2 Diabetes: Consequences for Motor Control, Musculoskeletal Function, and Whole-Body Movement

**DOI:** 10.1155/2013/508756

**Published:** 2013-02-17

**Authors:** Neil D. Reeves, Bijan Najafi, Ryan T. Crews, Frank L. Bowling

**Affiliations:** ^1^Institute for Biomedical Research into Human Movement and Health, School of Healthcare Science, Manchester Metropolitan University, Manchester M1 5GD, UK; ^2^Interdisciplinary Consortium on Advanced Motion Performance (iCAMP) and College of Medicine, The University of Arizona, Tucson, AZ 85724-5072, USA; ^3^Center for Lower Extremity Ambulatory Research (CLEAR), Rosalind Franklin University of Medicine and Science, North Chicago, IL 60064, USA; ^4^Faculty of Medical and Human Sciences, The University of Manchester, Manchester M13 9WL, UK

As highlighted by data in 2010 showing that 27% of US residents aged over 65 years have type 2 diabetes compared to 11% of people aged over 20 years, the risk of developing type 2 diabetes increases with advancing age. Accordingly, type 2 diabetes is predicted to rise concurrently with the increasing age of global populations. Diabetes causes a number of complications that negatively impact on the musculoskeletal system and the individual's capacity to perform a number of daily physical activities. It leads to impaired physical capacity through a number of mechanisms such as muscle weakness, limited joint range of motion, and damage to peripheral nerves (neuropathy). Persons affected tend to walk more slowly, with greater variability of gait, and are at increased risk of falling. Lower extremity complications are common, in particular 25% of diabetics develop a foot ulcer at some point. These difficult to heal ulcers commonly lead to amputation secondary to infection.

The vast majority of studies in diabetes patients evaluate gait within a laboratory setting. It may, however, be enlightening to study gait of diabetes patients in their natural environment where conditions may be different from those presented in the laboratory. E. D. de Bruin et al. present data showing the validity and reliability of a portable wearable sensor system for measuring gait parameters in an outdoor setting. They show that walking speed, cadence, step duration, and step length can be measured reliably in a challenging outdoor environment with diabetes patients. They further show that this portable system is able to discriminate between subgroups of diabetes patients with neuropathy based upon their step length.

Diabetic plantar ulcers develop predominantly due to high foot pressures applied during gait along with other risk factors such as neuropathy, vascular insufficiency, and foot deformities. When foot ulcers become infected, amputation may be considered the most appropriate course of action. In their paper, M. Tagoe and R. McCallum present a consecutive case series of their experience with transmetatarsal amputations in diabetic patients. This surgical procedure prevents further proximal spread of infection, whilst maximising limb function and maintaining a substantial portion of the foot. This procedure requires a sound understanding of functional anatomy via splitting and redirecting the tibialis anterior tendon to preserve an effective gait. 

Charcot foot is a devastating complication of diabetes with a twofold higher rate of major amputation compared to those without Charcot. The diagnosis can in fact be missed up to 95% of the time. A temperature difference (>2°C) between each foot can be indicative of a Charcot foot excluding other causes, but these measurements are typically taken at rest without considering the effect of plantar stress. B. Najafi et al. investigate a new approach for detecting temperature gradients between feet as a function of the number of steps walked. In this study B. Najafi et al. used a thermal imaging camera and custom analysis software to determine differences in plantar temperature after walking various distances and between diabetic groups with and without Charcot foot. They found that the thermal response to the graduated walking activity is a sensitive parameter to identify acute Charcot among patients with diabetes and peripheral neuropathy.

People with diabetes are weaker and have smaller muscles compared to matched controls without this condition, which will impact upon their ability to produce the required forces during activities of daily living. However, if the muscle area is infiltrated by noncontractile tissue, the muscle's force producing capability will be even lower than that estimated based upon its gross size. In their paper, L. J. Tuttle et al. measure the intramuscular adipose tissue present in the lower limb muscles of obese participants and diabetes patients with and without neuropathy. They show an increased ratio of intramuscular adipose tissue to muscle volume in the gastrocnemius compared to other lower limb muscles and also find negative correlations between various physical performance measures and calf muscle intramuscular adipose tissue/volume ratio.

Although muscle weakness is present in people with diabetes, skeletal muscle is remarkably adaptable. Resistance training programs are a well-established method for improving form and function, with various clinical and nonclinical populations showing increases in muscle size and strength after a period of training. N. Hovanec et al. perform a systematic review of the literature for the effects of resistance training on metabolic, neuromuscular, and cardiovascular function in older adults with type 2 diabetes. They find that resistance training can have a positive effect, with the largest effect found on the musculoskeletal system, benefits were also reported in aspects of the diabetic disease process, and to a lesser extent on changes in body composition.

In addition to being problems in their own right, complications from diabetes such as neuropathy, muscle weakness, foot and body pain, pharmacological complications, and specialty (offloading) footwear devices all contribute to individuals with diabetes being at higher risk of falling. The annual incidence of falls in the elderly with diabetes has been previously reported to be 39%. Furthermore, these individuals are at higher risk of fracture, have poorer rehabilitation results, and are at a higher risk of recurrent falls than their nondiabetic counterparts. Fortunately balance, strength, and gait training have been shown to successfully reduce fall risk in this population. The above issues relate to a “growing troubling triad” presented by diabetes, aging and falls and are reviewed in a paper by R. T. Crews et al. 

Diabetes and its associated complications present a number of challenges for motor control, musculoskeletal function, and whole-body movement. The high-quality collection of papers in this special issue furthers our understanding of these challenges. We hope that this special issue will inform and interest the reader and contribute to scientific understanding in this area.

